# Quality of orthodontic care—A multicenter cohort study in Germany: Part 1: Evaluation of effectiveness of orthodontic treatments and predictive factors

**DOI:** 10.1007/s00056-021-00304-3

**Published:** 2021-06-17

**Authors:** I. Graf, N. C. Bock, T. Bartzela, V. Röper, U. Schumann, K. Reck, H. Christ, K. Höfer, U. Fritz, D. Wiechmann, P.-G. Jost-Brinkmann, M. Wolf, S. Ruf, B. Braumann

**Affiliations:** 1 Department of Orthodontics, Faculty of Medicine and University Hospital of Cologne, Kerpener Str. 32, 50931 Cologne, Germany; 2 Department of Orthodontics, Faculty of Medicine and University Hospital of Gießen, University of Gießen, Gießen, Germany; 3 Institute of Dental and Craniofacial Sciences, Department of Orthodontics, Dentofacial Orthopedics and Pedodontics, Charité – Universitätsmedizin Berlin, Corporate Member of Freie Universität Berlin, Humboldt-Universität zu Berlin and Berlin Institute of Health, Berlin, Germany; 4 Department of Orthodontics, Hannover Medical School, Orthodontic Practice, Bad Essen, Germany; 5 Orthodontic Practice, Essen, Germany; 6 Orthodontic Practice, Pulheim, Germany; 7 Institute of Medical Statistics and Computational Biology (IMSB), University Hospital of Cologne, Cologne, Germany; 8 Department of Operative Dentistry and Periodontology, Faculty of Medicine and University Hospital of Cologne, University of Cologne, Cologne, Germany; 9 Department of Orthodontics, Faculty of Medicine and University Hospital RWTH Aachen, RWTH Aachen, Aachen, Germany

**Keywords:** Malocclusion, Treatment outcome, Predictive factors, Dental health services, Malokklusion, Behandlungsergebnis, Prädiktive Faktoren, Versorgungsforschung

## Abstract

**Aims:**

Orthodontic care and its effectiveness have increasingly become the focus of political and public attention in the recent past. Therefore, this multicenter cohort study aimed to report about the effectiveness of orthodontic treatments in Germany and to identify potential influencing factors.

**Methods:**

A total of 586 patients from seven German study centers were screened for this cohort study, of which 361 patients were recruited at the end of their orthodontic treatment. Of these, 26 patients had missing study models and/or missing treatment information. Thus, 335 participants were included. The severity of malocclusion was rated using the Peer Assessment Rating (PAR) Index at baseline (T0) retrospectively and—prospectively—after the retention period (T1). Practitioner-, treatment- and patient-related information were analyzed in order to detect potential predictive factors for treatment effectiveness.

**Results:**

Study participants (202 female and 133 male) were on average 14.8 (standard deviation [SD] ± 6.1) years old at start of active treatment. Average PAR score at T0 was 25.96 (SD ± 10.75) and mean posttreatment PAR score was 3.67 (SD ± 2.98) at T1. An average decrease of total PAR score by 22.30 points (SD ± 10.73) or 83.54% (SD ± 14.58; *p* < 0.001) was detected. Furthermore, 164 treatments (49.1%) were categorized as ‘greatly improved’ but only 3 treatments (0.9%) as ‘worse or no different’; 81.5% of all cases finished with a high-quality treatment outcome (≤5 PAR points at T1). Logistic regression analyses detected staff experience as a significant predictive factor for high-quality results (odds ratio 1.27, *p* = 0.001, 95% confidence interval 1.11–1.46).

**Conclusion:**

The improvement rate among this selected German cohort indicated an overall very good standard of orthodontic treatment. Staff experience proved to be a predictive factor for high-quality results.

## Abstract (de)

**Ziel:**

Die kieferorthopädische Versorgung und ihre Effektivität sind in der jüngeren Vergangenheit zunehmend in den Fokus von Politik und Öffentlichkeit gelangt. Ziel dieser multizentrischen Kohortenstudie war es daher, Erkenntnisse über die Effektivität von kieferorthopädischen Behandlungen in Deutschland zu generieren und mögliche Einflussfaktoren zu erfassen.

**Methoden:**

Für diese Kohortenstudie wurden insgesamt 586 Patient*innen aus sieben deutschen Studienzentren gescreent, von denen 361 Patient*innen bei Abschluss ihrer kieferorthopädischen Behandlung rekrutiert werden konnten. Von diesen fehlten bei 26 Patient*innen Studienmodelle und/oder Behandlungsinformationen. Daher wurden 335 Teilnehmende in die Studie aufgenommen. Der Schweregrad der Malokklusion wurde mit dem PAR(Peer Assessment Rating)-Index zu Beginn (T0) retrospektiv und – nach der Retentionszeit (T1) – prospektiv bewertet. Behandler-, therapie- und patient*innenbezogene Informationen wurden analysiert, um mögliche prädiktive Faktoren für die Wirksamkeit der Behandlung zu ermitteln.

**Ergebnisse:**

Die Probanden (202 weibliche, 133 männliche) waren zu Beginn der aktiven Behandlung im Durchschnitt 14,8 (Standardabweichung [SD] ± 6,1) Jahre alt. Der durchschnittliche PAR-Score zum Zeitpunkt T0 lag bei 25,96 (SD ± 10,75), der durchschnittliche PAR-Score nach der Behandlung bei 3,67 (SD ± 2,98; T1). Dabei wurde eine mittlere PAR-Score-Reduktion von 22,30 Punkten (SD ± 10,73) oder 83,54 % (SD ± 14,58; *p* < 0,001) festgestellt. Darüber hinaus wurden 164 Behandlungen (49,1 %) als „greatly improved“ kategorisiert, aber nur 3 Behandlungen (0,9 %) als „worse or no different“; 81,5 % aller Fälle schlossen mit einem hochwertigen okklusalen Behandlungsergebnis (≤5 PAR-Punkte bei T1) ab. Logistische Regressionsanalysen wiesen die Erfahrung des an der Behandlung beteiligten Personals als signifikanten prädiktiven Faktor für qualitativ hochwertige Ergebnisse nach (Odds Ratio 1,27, *p* = 0,001, 95 %-Konfidenzintervall 1,11‑1,46).

**Zusammenfassung:**

Die okklusale Verbesserungsquote in dieser ausgewählten deutschen Kohorte ließ auf einen insgesamt sehr hohen Standard der kieferorthopädischen Behandlung schließen. Die Erfahrung des behandelnden Teams erwies sich als ein prädiktiver Faktor für qualitativ hochwertige Ergebnisse.

## Introduction

Orthodontic care and especially its effectiveness have increasingly become the focus of political and public attention in the recent past. In 2018, the German Federal Ministry of Health commissioned an evaluation about orthodontic treatments and their potential influence on oral health by the Institute for Health and Social Research (Institut für Gesundheits- und Sozialforschung, IGES). The IGES report came to the conclusion that the dental health benefits of orthodontic treatments currently lack evidence which in turn is no proof against such benefits [15]. Moreover, oral health not only comprises dental health aspects like tooth loss or caries, but also revolves around functional, emotional, and social issues. In this context, oral health-related quality of life has proven to be reduced in children, adolescents and adults with specific malocclusions [1, 23, 38]. Dental appearance might have significant psychosocial effects. As our population becomes increasingly more aware of dental appearance and is highly informed about orthodontic treatment opportunities, the general demand for orthodontic treatment has risen [17, 20]. According to the German Health Interview and Examination Survey for Children and Adolescents (KiGGS Wave 2) by the Robert Koch Institute with a cohort of 15,023 children and adolescents, 25.8% of all 3‑ to 17-year-old girls and 21.1% of all 3‑ to 17-year-old boys were in active orthodontic treatment between the years 2014 and 2017. During this time span, 13-year-old girls and 14-year-old boys underwent orthodontic treatment most frequently (55.0% and 50.8%, respectively). The authors mentioned an increase in uptake of orthodontic care over the past decade [35]. Population-based data about orthodontic treatment in Germany along with its outcome and effectiveness are however lacking.

Generally, measuring treatment outcome and effectiveness have been discussed thoroughly in international orthodontic literature. In order to assess the severity and complexity of malocclusions before and after orthodontic treatment, numerous grading systems have been proposed [6, 7, 24, 31]. However, currently no internationally recognized and consistently used quality assessment tool exists. One of the many systems is the Peer Assessment Rating (PAR) Index developed by Richmond et al. to provide an objective assessment of treatment success [32]. The PAR Index is an occlusal index that is able to quantitatively evaluate orthodontic treatment outcome by measuring pre- and posttreatment models and the respective improvement rate. It has shown excellent validity and reliability [31, 36]. When reporting about effectiveness and treatment outcome using the PAR Index, some authors suggest to not solely report about the change between pre- and posttreatment PAR scores, but rather take the final total PAR score as an indicator for a good occlusal outcome. Improvement rates seem to be less sensitive because of the confounding factor of the pretreatment PAR score [30].

The outcome of orthodontic treatment might be influenced by specific patient-, practitioner-, and treatment-related factors [21, 30]. There is varying evidence which patient gender or type of malocclusion is associated with better occlusal outcomes [5, 19, 41, 42]. Treatment-related factors like the type of appliance (fixed vs. removable) or the number of arches treated (single vs. dual arch treatment) were part of corresponding research as well [25, 33, 39]. Although international study groups have previously reported about orthodontic treatment outcome and potential influencing factors in large cohorts and for a variety of treatment modalities [16, 30], such research about orthodontic reality in Germany is scarce and often involves patients of only one or two orthodontic providers/university hospitals [21, 40].

In the light of the above-mentioned need for national research, the aim of this explorative multicenter cohort study was to evaluate the effectiveness of orthodontic treatments in Germany as well as potential predictive factors within this cohort.

## Subjects and methods

### Study centers, study participants, and recruitment procedure

This multicenter cohort study was approved by all corresponding ethics committees with the leading one being the Ethics Committee of the University Hospital of Cologne (#14-425).

Most study centers were asked to participate in this cohort study by the principal investigator (IG), based on existing research connections and structures. Thus, study center selection was not random, but the research objectives were transparently communicated prior to study start through various ways (e.g., e‑mail to all delegates of the DGKFO [Deutsche Gesellschaft für Kieferorthpädie e. V.], announcements at national meetings of university professors, informal information transfer), so potentially every orthodontic practice/university hospital in Germany had the opportunity to join the project.

Prior to recruitment, study centers were informed about the details of the recruitment procedure. Each study center designated one investigator in charge. All upcoming posttreatment record takings were to be screened and the corresponding patients as well as their legal guardians to be informed about the study. In Germany, posttreatment records (e.g., impressions, x‑rays, photographs) usually mark the end of an orthodontic treatment and are frequently taken after a certain period of retention time after active orthodontic treatment (e.g., after bracket removal). Prior to study start, we assumed this period of time to be about one year. Inclusion criteria were the following: orthodontically treated patients ≥11 years of age at the end of their orthodontic treatment, prior to posttreatment record taking. Patients with severe systemic diseases, immunosuppression and/or syndromes were excluded from this study. Signed informed consent was mandatory. Patient-related data were pseudonymized. Through detailed screening logs and repeated monitoring of the recruitment process by the principal investigator (IG) possible patient selection bias should be limited. The recruitment and monitoring procedures were defined with the Clinical Trials Center of the University Hospital of Cologne prior to study start.

### Assessment of treatment characteristics

In order to report about specific treatment characteristics, all study centers were asked to provide information about the indication to treat according to German KIG criteria (= Kieferorthopädische Indikationsgruppen, German index of treatment need) [12], early orthodontic treatment (EOT), the duration of active treatment and the appliances and treatment modalities used. Treatment modalities considered in this study were the following: removable appliances (RA), multibracket appliances (MBA), fully individualized lingual multibracket appliances (L-MBA), Herbst appliances (HA), rapid maxillary expansion (RME), and orthognathic surgery (OS).

Since this study aimed to evaluate a variety of aspects of the quality of orthodontic care in Germany, we also included performer-specific variables, i.e., staff experience in years and treatment at a university hospital/private orthodontic practice.

### Assessment of occlusal characteristics

Pretreatment study models (T0) were obtained from the archives of the study centers, while posttreatment study models (T1) were provided prospectively after impression taking. Occlusal characteristics were measured by using the PAR Index according to the British weighting system [9]. The principal investigator (IG)—a PAR-certified, calibrated, and experienced examiner—was in charge of the measurements for six of the seven participating study centers. Because of potential bias due to the respective affiliation of IG, a second PAR-certified, calibrated and highly experienced examiner (NCB) performed measurements for one of the seven study centers. Treatments were categorized as ‘improved’ if a PAR score reduction of at least 30% was achieved. A change of at least 22 PAR points stood for ‘great improvement’. An improvement rate of less than 30% was declared as ‘worse or no different’. According to Richmond et al., a total PAR score reduction of at least 70% within a sample stands for good quality of care and less than 5% of all cases should be categorized as ‘worse or no different’ [32]. A final PAR score of ≤5 points stands for an almost ‘ideal occlusion’ and a high-quality treatment result; a final score of ≤10 points for an acceptable occlusion [30]. By applying both types of analyses—PAR score reduction and final PAR score—we were able to discuss the quality of the course of treatments (= PAR score reduction) as well as the quality of the final results (= final PAR score).

## Statistical analyses

Due to the exploratory nature of this study, no sample size calculation was performed. We considered previous studies [3, 11, 29, 37] and aimed for a similar sample size, yet considering the wider range of patient-, practitioner- and treatment-related factors involved in our cohort study. A sample size of 60 patients per study center appeared to be sufficient.

Reliability testing was performed by evaluating *intra*examiner (IG) and *inter*examiner reliability (IG vs. NCB) using the intraclass correlation coefficient (ICC) for total PAR scores at T0 and T1. Furthermore, 20% of the study models at one of the study centers were randomly selected and rescored by the principal investigator (IG) after a 30-day period in relation to the first scoring. Finally, all cases scored by NCB were additionally scored by IG to test for interexaminer reliability.

Our primary endpoint was the weighted PAR score reduction between T0 and T1. Because our data failed the Shapiro–Wilk normality test, nonparametric tests were performed. For continuous variables, descriptive statistics (mean, standard deviation, minimum, 1st quartile, median, 3rd quartile and maximum) were calculated and compared by the Wilcoxon test. Qualitative variables were summarized by count and percentage, and their influential impact was analyzed by using crosstabs in combination with Pearson’s chi-square and Fisher’s exact test. Binary logistic regression helped to determine predictive factors of final PAR score (≤5). Independent variables that indicated statistical significance and/or clinical significance in crosstab analyses were tested in this model. Odds ratios (OR) and 95% confidence intervals (CI) were provided for potential influencing factors.

Statistical analyses were performed with SPSS® statistical package (version 23, IBM, Armonk, NY, USA), in cooperation with the Institute of Medical Statistics and Computational Biology of the University Hospital of Cologne. A two-sided *p*-value of less than 0.05 was considered to indicate statistical significance. No adjustment for multiple testing was performed; thus, all analyses, except those related to the primary endpoint, were considered to be exploratory.

## Results

### Recruitment of study participants

**Fig. 1:**
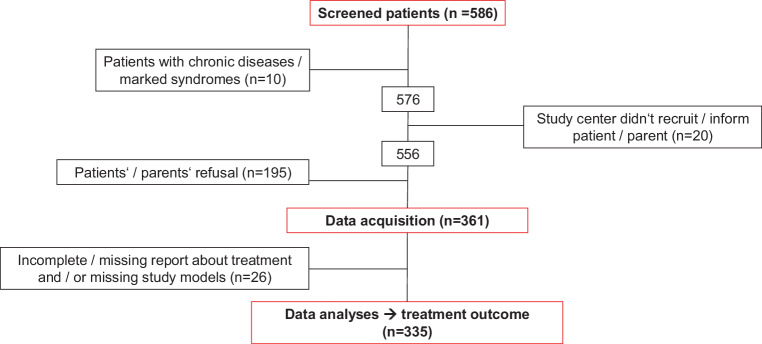
Flow chart of patient enrollment Flussdiagramm der Patient*innenrekrutierung

A total of 586 patients from seven German study centers—four university hospitals and three orthodontic practices—were screened (Fig. 1). Screening and recruitment did not start simultaneously at all study centers and lasted between 5 and 17 months between 2016 and 2019 (Table 1). The recruited sample comprised 361 patients, of which 335 patients could be included in the analyses because 26 patients had missing study models and/or missing treatment information. Final recruitment rate varied between 31.5 and 78.4% (mean 57.2%, Table 1); drop-out and/or exclusion reasons are shown in the participant flow chart (Fig. 1).

**Table 1:** Descriptive information about study centers with regards to months of effective recruitment, patients included and screened (recruitment rate in parentheses), percentage PAR change (T0–T1 mean; standard deviation), duration between end of active orthodontic treatment and recruitment (= posttreatment record taking), number of staff members involved as well as explanation about the respective years of orthodontic experience; orthodontic experience was counted as being “0” for postgraduates Deskriptive Informationen zu den Studienzentren: Dauer der effektiven Rekrutierung in Monaten, Anzahl gescreenter und eingeschlossener Patient*innen (Rekrutierungsrate in Klammern), prozentuale PAR-Wert-Verbesserung (T0–T1, Mittelwert mit Standardabweichung), Zeitraum zwischen Ende der aktiven kieferorthopädischen Behandlungsphase (z. B. Entfernung Multibracketapparatur) und Rekrutierung (Abschlussdiagnostik) in Monaten, Anzahl der beteiligten Behandler*innen und deren kieferorthopädische Erfahrung in Jahren; kieferorthopädische Erfahrung wurde bei Weiterbildungsassistent*innen mit “0” berechnet

	Effective months of recruitment	Patients included/patients screened (%)	% PAR change T0-T1	Mean months between end of active orthodontic treatment and recruitment	Staff members (O; P)	Staff experience (in years; range of O experience)
Practice 1	5	60/91 (65.9)	90.62 (SD ± 12.3)	6.3	5 (4 O, 1 P)	5.4 (1–20)
Practice 2	13	23/73 (31.5)	81.80 (SD ± 10.3)	16.6	5 (3 O, 2 P)	12.8 (5–39)
Practice 3	17	66/97 (68.0)	87.74 (SD ± 8.8)	19.1	5 (4 O, 1 P)	8.8 (4–25)
–	–	–	–	Mean 14.0	–	Mean 9.0
University hospital 1	10	58/74 (78.4)	76.29 (SD ± 16.9)	13.3	10 (2 O, 8 P)	1.5 (1–14)
University hospital 2	6	13/40 (32.5)	81.80 (SD ± 10.7)	15.6	9 (3 O, 6 P)	4.4 (1–26)
University hospital 3	8	59/118 (50.0)	84.72 (SD ± 14.1)	26.0	9 (4 O, 5 P)	5.3 (4–22)
University hospital 4	17	56/93 (60.2)	77.60 (SD ± 17.4)	14.4	8 (4 O, 4 P)	4.3 (1–24)
–	–	–	–	Mean 17.3	–	Mean 3.9
Total/Mean	11	335/586 (57.2)	83.54 (SD ± 14.6)	15.9	–	6.1

*O* orthodontist, *P* postgraduate, *SD* standard deviation, *PAR* Peer Assessment Rating Index

### Practitioner characteristics

**Fig. 2:**
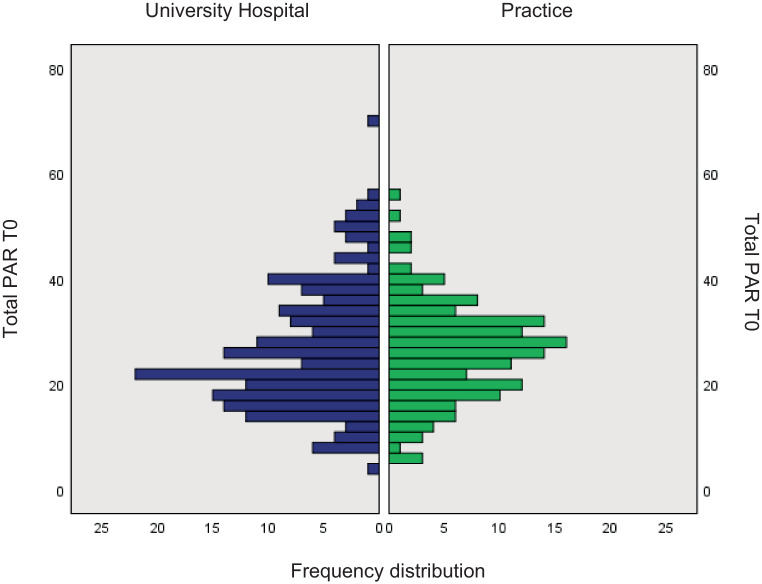
Histogram of the distribution of weighted total mean Peer Assessment Rating (PAR) Index score at T0 at university hospitals versus at orthodontic practices; no significant difference detected (*p* = 0.520) Histogramm zur Verteilung der mittleren gewichteten PAR(Peer Assessment Rating)-Werte zu T0 an Universitätskliniken im Vergleich zu Praxen; kein statistisch signifikanter Unterschied ermittelt (*p* = 0,520)

Descriptive information about all study centers and their recruitment is shown in Table 1. The duration of effective recruitment had no effect on the respective treatment outcome within each study center. More postgraduates worked at university hospitals compared to orthodontic practices, which resulted in significantly higher mean staff experience (SE) in years at practices (mean SE 9.0 vs. 3.9 years, respectively; Table 1). There was no significant difference between the initial PAR score (T0) of cases started at an orthodontic practice and those started at a university hospital (mean PAR score 25.83 vs. 26.07, respectively, *p* = 0.520; Fig. 2). Active treatments lasted significantly longer at orthodontic practices than treatments at university hospitals (36.9 vs. 26.8 months, *p* < 0.001; Table 2), while the retention period was slightly shorter in orthodontic practices (14.0 vs. 17.3 months; Table 1).

**Table 2:** Treatment characteristics Deskriptive Darstellung behandlungsspezifischer Parameter

	*n*^a^	%	Active treatment duration (months ± SD)
Patients total	335	100	–
Missing information	3	–	–
–	332	–	31.3 (± 16.1)
University hospital	186	55.5	26.8 (± 13.7)
Orthodontic practice	149	45.5	36.9 (± 17.0)
MBA total	327	97.61	26.2 (± 11.8)
MBA only	246	73.4	26.1 (± 12.3)
RA only	5	1.5	28.4 (± 9.8)
RA plus MBA	81	24.2	44.6 (± 15.6)
RA total	86	25.7	–
Active appliances (upper/lower)	31	36.0	–
Functional appliances	53	61.6	–
Extraoral devices	1	1.2	–
Missing information	1	1.2	–
L‑MBA	52	15.5	–
HA	42	12.5	–
RME	42	12.5	–
OS	20	6.0	–
EOT	30	9.0	–

*SD* standard deviation; *MBA* multibracket appliance, *RA* removable appliance, *L‑MBA* fully individualized lingual multibracket appliance, *HA* Herbst appliance, *RME* rapid maxillary expansion, *OS* orthognathic surgery, *EOT* early orthodontic treatment

^a^*n* values might differ from the total number of patients (*n* = 335) because of missing information regarding treatment duration/beginning of treatment

### Patient characteristics

There were 202 female patients (60.3%) and 133 male patients (39.7%). Study participants were 14.8 years old on average (SD ± 6.1) at active treatment start.

In some patients, individual patient and treatment characteristics could not be determined. This was the case if, for example, patients changed their initial orthodontist. Therefore, some of the *n* values differ from the total number of patients of whom we used the data for the presented analyses.

### Treatment characteristics

**Fig. 3:**
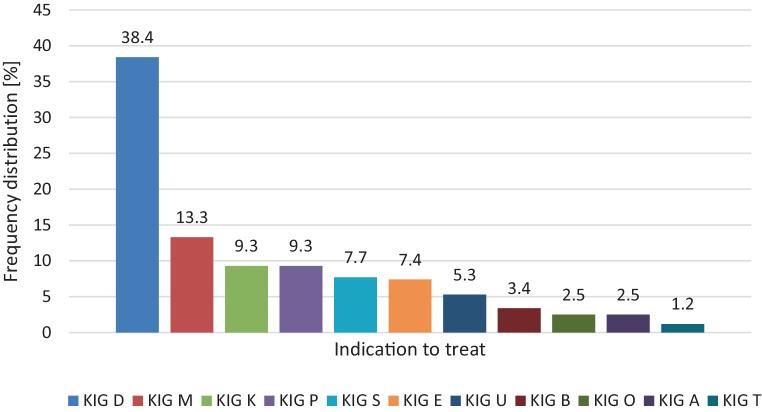
Indication to treat according to KIG (Kieferorthopädische Indikationsgruppen, German index of treatment need) criteria in percent. *KIG A* craniofacial anomaly, *KIG B* transverse discrepancy, scissor bite, *KIG D* enlarged overjet, *KIG E* contact point displacement, crowding, *KIG K* transverse discrepancy, crossbite bilateral/unilateral, *KIG M* negative overjet ≤0 mm, *KIG O* open bite, *KIG P* crowding in the posterior area, *KIG S* impacted tooth, *KIG T* enlarged overbite, *KIG U* missing tooth; private treatments not covered by public insurance were accounted for with their KIG equivalent Behandlungsindikation gemäß KIG(kieferorthopädische Indikationsgruppen)-Schema in Prozent; *KIG A* kraniofaziale Anomalie, *KIG B* transversale Abweichung, bukkale Nonokklusion, *KIG D* vergrößerte sagittale Frontzahnstufe, *KIG E* Kontaktpunktabweichung, Engstand, *KIG K* transversale Abweichung, Kopfbiss, ein-/beidseitiger Kreuzbiss, *KIG M* reduzierte sagittale Frontzahnstufe ≤0 mm, *KIG O* offener Biss, *KIG P* Platzmangel im Seitenzahnbereich/in der Stützzone, *KIG S* Durchbruchsstörung, *KIG T* Tiefbiss; *KIG U* Unterzahl bleibender Zähne; sog. Privatbehandlungen, deren Kosten nicht von gesetzlichen Krankenversicherungen übernommen wurden, wurden mit dem jeweiligen KIG-Äquivalent in die Analyse einbezogen

The distribution of the indication to treat according to KIG criteria is shown in Fig. 3. Note that 16.7% of all treatments were not covered by public insurance. Yet, we accounted for their initial KIG equivalent and included these in Fig. 3. The majority of patients within this sample were treated with fixed appliances (97.6%, *n* = 327, B‑MBA plus L‑MBA), either in combination with or without other devices. In all, 25.7% of all treatments (*n* = 86) consisted of a phase with RA, while 5 treatments involved RA only (Table 2). Looking at the distribution in detail, we found that 46.3% of patients at an orthodontic practice, but only 18.3% of patients at a university hospital were treated with both removable and fixed appliances. This difference was significant (*p* < 0.001).

Treatment characteristics in relation to age of the patients at active treatment start is shown in Table 3.

**Table 3:** Treatment characteristics in relation to pretreatment age Behandlungsspezifische Parameter in Relation zum Alter bei Behandlungsstart

	*n*	Age Total treatment (years)	*p*-value^a^	*n*	Age RA ± MBA (years)	*p*-value^a^	*n*	Age MBA only (years)	*p*-value^a^
Total mean	332	14.8 (SD ± 6.1)	–	86	11.4 (SD ± 1.9)	–	246	16.0 (SD ± 6.6)	–
OS	20	24.7 (SD ± 9.9)	**<0.001**	0	–	–	20	24.7 (SD ± 9.9)	**<0.001**
RME	42	15.2 (SD ± 7.2)	0.983	11	9.6 (SD ± 2.0)	**0.002**	31	17.2 (SD ± 7.3)	0.198
HA	42	14.8 (SD ± 3.0)	**0.009**	6	11.0 (SD ± 2.2)	0.672	36	15.4 (SD ± 2.6)	0.094
L‑MBA	52	17.1 (SD ± 8.3)	**<0.001**	21	12.7 (SD ± 1.2)	**<0.001**	31	20.2 (SD ± 9.7)	**<0.001**
Orthodontic practice	147	14.4 (SD ± 6.7)	**0.005**	69	11.5 (SD ± 1.8)	0.266	78	16.9 (SD ± 8.3)	0.364
University hospital	185	15.1 (SD ± 5.5)		17	11.0 (SD ± 2.0)		168	15.5 (SD ± 5.6)	
*PAR Index* *Reduction category*									
Worse or no different	3	12.5 (SD ± 0.6)	**0.035**	1	12.6 (SD ± 0.0)	0.351	2	12.4 (SD ± 0.8)	0.775
Improved	166	15.1 (SD ± 5.7)		30	11.6 (SD ± 1.9)		136	15.9 (SD ± 6.0)	
Greatly improved	163	14.5 (SD ± 6.5)		55	11.3 (SD ± 1.9)		108	16.2 (SD ± 7.4)	
Ideal occlusion	271	14.6 (SD ± 5.9)	0.334	79	11.4 (SD ± 1.8)	0.630	192	15.9 (SD ± 6.5)	0.807

*SD* standard deviation, *PAR index* Peer Assessment Rating Index, *MBA* multibracket appliance, *RA* removable appliance, *L‑MBA* fully individualized lingual multibracket appliance, *HA* Herbst appliance, *RME* rapid maxillary expansion, *OS* orthognathic surgery, *EOT* early orthodontic treatment

^a^*p*-value in relation to those patients who did not receive the specific treatment modality; Fisher’s exact test;* bold* values statistically significant at 5%

### Treatment outcome

Both intra- and interexaminer reliability for PAR score measurements were excellent (ICCs > 0.900; Table 4). The average PAR score at T0 was 25.96 (SD ± 10.75). A significant decrease of the total PAR score by 22.30 points (83.54%) was detected (T0–T1: 25.96 vs. 3.67; *p* < 0.001; Table 5). Assessing the different components that make up the total PAR score, each component showed a significant improvement after active treatment compared to baseline (Table 5). The smallest improvement rate was found within the PAR component ‘centerline’ (T0–T1: 1.60 vs. 0.20; 33.23% reduction) followed by ‘buccal segments’ (T0–T1: 4.40 vs. 2.13; 44.02% reduction), yet these improvement rates were still found to be statistically significant.

**Table 4:** Intraclass correlation coefficient (ICC) for intraexaminer reliability (IG) and interexaminer reliability (IG vs. NCB) testing Intraklassenkorrelationskoeffizient (ICC) für Intrarater- (IG) und Interrater-Reliabilität (IG vs. NB)

	ICC	95% CI	
		Lower limit	Upper limit
*IG*			
PAR T0	0.997	0.989	0.999
PAR T1	0.988	0.958	0.996
*IG vs. NCB*			
PAR T0	0.998	0.997	0.999
PAR T1	0.936	0.890	0.962

*PAR T0* total weighted Peer Assessment Rating Index score at T0; *PAR T1* total weighted Peer Assessment Rating Index score at T1, *95% CI* 95% confidence interval

**Table 5:** Mean weighted components and total PAR score before (T0) and after treatment (T1); *n* = 335; *SD* standard deviation, *in* *parentheses* minimum–maximum Mittlere gewichtete PAR-Werte vor (T0) und nach kieferorthopädischer Behandlung (T1); *n* = 335; *SD* Standardabweichung, *in Klammern* Minimum und Maximum

	Before (T0)	After treatment (T1)	Point change T0–T1	% change T0–T1	*p*^a^ (T0 vs. T1)
Upper anterior segments	4.46 (SD ± 3.0; 0–15)	0.17 (SD ± 0.6; 0–8)	4.29 (SD ± 3.0; 0–15)	90.94 (SD ± 25.1; 0–100)	**<0.001**
Lower anterior segments	2.72 (SD ± 2.6; 0–13)	0.22 (SD ± 0.6; 0–3)	2.50 (SD ±2.6; −1 to 12)	73.59 (SD ± 42.2; −100 to 100)	**<0.001**
Buccal segments	4.40 (SD ± 2.1; 1–12)	2.13 (SD ± 1.3; 0–8)	2.27 (SD ± 2.1; −3 to 10)	44.02 (SD ± 36.2; −100 to 100)	**<0.001**
Overjet	10.08 (SD ± 7.4; 0–36)	0.41 (SD ± 1.5; 0–6)	9.67 (SD ± 7.4; −6 to 36)	78.59 (SD ± 39.7; 0–100)	**<0.001**
Overbite	2.66 (SD ± 1.7; 0–8)	0.50 (SD ± 0.9; 0–4)	2.16 (SD ± 1.7; −4 to 8)	72.10 (SD ± 41.3; 0–100)	**<0.001**
Centerline	1.60 (SD ± 2.3; 0–8)	0.20 (SD ± 0.9; 0–8)	1.40 (SD ± 2.4; −4 to 8)	33.23 (SD ± 47.0; 0–100)	**<0.001**
PAR total	25.96 (SD ± 10.8; 4–70)	3.67 (SD ± 3.0; 0–21)	22.30 (SD ± 10.7; 1–68)	83.54 (SD ± 14.6; 14–100)	**<0.001**

*PAR *Peer Assessment Rating Index

^a^ Wilcoxon test; *bold* values represent statistical significance at 5%

### ‘Great improvement’ and predictive factors

A total of 168 patients (50.1%) could be allocated to the ‘improved’ category; 164 treatments (49.0%) were categorized as ‘greatly improved’. More females than males were treated with ‘great improvement’ (56.7 vs. 43.3%, *p* = 0.219). Patients were slightly younger at treatment start and treatment duration was longer in the ‘greatly improved’ group than in the ‘improved’ group (14.5 vs. 15.1 years, *p* = 0.035; 34.7 vs. 27.8 months, *p* < 0.001 respectively; Table 6).

**Table 6:** Summary of univariate analyses through crosstabs of potential predictive factors for ‘greatly improved’ vs. ‘improved’ vs. ‘worse or no different’ treatments Zusammenfassung der univariaten Analysen durch kreuztabellarische Darstellung potenzieller prädiktiver Faktoren für die PAR-Kategorien „greatly improved“ vs. „improved“ vs. „worse or no different“

	Greatly improved *n* (%)	Improved *n* (%)	Worse or no different *n* (%)	*p*-value^a^
*Total mean*	164 (49.0)	168 (50.1)	3 (0.9)	–
Female	93 (46.0)	106 (52.5)	3 (1.5)	0.210
Male	71 (53.4)	62 (46.6)	0 (0.0)	
Age at active treatment start	14.5 years	15.1 years	12.5 years	**0.035**
Total treatment duration	34.7 months	27.8 months	37.3 months	**<0.001**
*EOT*				
Yes	17 (56.7)	13 (43.3)	0 (0.0)	0.583
No	147 (48.2)	155 (50.8)	3 (1.0)	
*RA*				
Yes	61 (59.2)	42 (40.8)	0 (0.0)	**0.026**
No	103 (44.4)	126 (54.3)	3 (1.3)	
*OS*				
Yes	16 (80.0)	4 (20.0)	0 (0.0)	**0.017**
No	148 (47.1)	163 (51.9)	3 (1.0)	
*RME*				
Yes	33 (78.6)	9 (21.4)	0 (0.0)	**<0.001**
No	131 (44.7)	159 (54.3)	3 (1.0)	
*HA*				
Yes	26 (61.9)	16 (38.1)	0 (0.0)	0.180
No	138 (47.3)	151 (51.7)	3 (1.0)	
*L‑MBA*				
Yes	29 (55.8)	23 (44.2)	0 (0.0)	0.619
No	135 (47.9)	144 (51.1)	3 (1.1)	
*Mean SE 9.0 years*	86 (57.7)	62 (41.6)	1 (0.7)	**0.009**
*Mean SE 3.9 years*	78 (41.9)	106 (57.0)	2 (1.1)	

*SE* staff experience, *RA* removable appliance, *L‑MBA* fully individualized lingual multibracket appliance, *HA* Herbst appliance, *RME* rapid maxillary expansion, *OS* orthognathic surgery, *EOT* early orthodontic treatment

^a^Fisher’s exact test; *bold* values represent statistical significance at 5%

In all, 55.8% of all L‑MBA cases were categorized as ‘greatly improved’ (*n* = 29, *p* = 0.619); 61.9% of all HA treatments classified for ‘great improvement’ (*n* = 61.9, *p* = 0.180). A significantly greater number of patients treated with RA, OS as well as treated with RME showed ‘great improvement’ at T1 in comparison to those who had not been treated with the mentioned treatment modalities (59.2%, *n* = 61, *p* = 0.026; 80%, *n* = 16, *p* = 0.017; 78.6%, *n* = 33, *p* < 0.001 respectively). EOT had no significant influence on the improvement rate (*p* = 0.583; Table 6).

Significantly more treatments resulted in ‘great improvement’ when treated with high mean SE compared to cases treated with low mean SE in years (57.7%, *n* = 86 vs. 41.9%, *n* = 78; *p* = 0.009; Table 6). Especially when looking at the different PAR components in detail, the following difference emerged: The PAR component ‘buccal segments’ was reduced by 36.92% when treated with low SE and by 52.88% when treated with high SE (*p* < 0.001). Yet, even when treated with low mean SE, cases proved to be of a good standard with 41.9% being ‘greatly improved’, 57.0% ‘improved’ and only 1.1% ‘worse or no different’.

### High-quality treatment result and predictive factors

A total of 81.5% of patients (*n* = 273) finished with a high-quality treatment result with a final PAR score ≤5; 96.4% of all patients (*n* = 323) had an acceptable result with a final PAR score ≤10 (Table 7).

**Table 7:** Summary of univariate analyses through crosstabs of potential predictive factors for high-quality results with final PAR scores ≤5 Zusammenfassung der univariaten Analysen durch kreuztabellarische Darstellung potenzieller prädiktiver Faktoren für hohe Ergebnisqualität mit finalen PAR-Werten ≤5

	High-quality result with final PAR score ≤5 *n* (%)	Final PAR score >5 *n* (%)	*p*-value^a^
Total mean	273 (81.5)	62 (18.5)	–
Female	167(82.7)	35 (17.3)	0.566
Male	106 (79.7)	27 (20.3)	
Age at active treatment start	14.6 years	15.6 years	0.334
Total treatment duration	31.9 months	28.6 months	0.322
*EOT*			
Yes	24 (80.0)	6 (20.0)	0.807
No	249 (81.6)	56 (18.4)	
*RA*			
Yes	93 (90.3)	10 (9.7)	**0.006**
No	180 (77.6)	52 (22.4)	
*OS*			
Yes	13 (65.0)	7 (35.0)	0.067
No	260 (82.8)	54 (17.2)	
*RME*			
Yes	28 (66.7)	14 (33.3)	**0.010**
No	245 (83.6)	48 (16.4)	
*HA*			
Yes	34 (81.0)	8 (19.0)	0.833
No	239 (81.8)	53 (18.2)	
*L‑MBA*			
Yes	50 (96.2)	2 (3.8)	**0.002**
No	223 (79.1)	59 (20.9)	
Mean SE 9.0 years	136 (91.3)	13 (8.7)	**<0.001**
Mean SE 3.9 years	137 (73.7)	49 (26.3)	

*SE* staff experience, *RA* removable appliance, *L‑MBA* fully individualized lingual multibracket appliance, *HA* Herbst appliance, *RME* rapid maxillary expansion, *OS* orthognathic surgery, *EOT* early orthodontic treatment, *PAR *Peer Assessment Rating Index

^a^Fisher’s exact test; *bold* values represent statistical significance at 5%

A significantly high number of patients with L‑MBA had an almost ‘ideal occlusion’ (96.2%, *n* = 50, *p* = 0.002) compared to those treated otherwise. Looking at the different components of the PAR Index, we detected a statistically significant difference in the component ‘buccal segments’ between lingually treated patients and the rest as this PAR component improved significantly more in patients with L‑MBA treatments (mean PAR score T1 1.25 vs. 2.30, 66.89% vs. 39.84% reduction within this PAR component, *p* < 0.001).

Of treatments with a RA phase (mostly functional appliances), 90.3% (*n* = 93) resulted in high-quality treatment results, which were significantly more than treatments without RA (*p* = 0.006). Furthermore, 65% (*n* = 13) of all patients who underwent OS finished with a final PAR score ≤5 (*p* = 0.067). Combined orthodontic–orthognathic surgery treatment lasted longer and initial PAR score was significantly higher than in all other cases (28.8 vs. 26.5 months, *p* = 0.245; mean initial PAR T0 36.50 vs. 25.29, *p* < 0.001 respectively). Of all patients with RME, 66.7% (*n* = 28) had an almost ‘ideal occlusion’ at the end of their treatments. This was significantly less than the distribution among the rest of the patients (*p* = 0.010). Finally, 81.0% (*n* = 34) of all HA treatments finished with an end PAR score ≤5 (*p* = 0.833). High-quality treatment results were significantly more frequent when treated with high mean SE compared to low mean SE in years (91.3%, *n* = 136 vs. 73.7%, *n* = 137; *p* < 0.001; Table 7).

Table 8 shows logistic regression analyses with ‘high-quality treatment outcome’ (final PAR score ≤5) as the dependent variable. Staff experience proved to be a significant predictive factor for high-quality treatment results (OR 1.27, *p* = 0.001). Patients who received RA—in addition to fixed appliances—had a 1.92 higher chance to finish with a PAR score ≤5, yet this can only be regarded as a trend because of missing statistical significance (*p* = 0.089). Note that the majority of RA treatments consisted of functional appliances (Table 2). It was less likely to finish with a high-quality result in treatments with RME (OR 0.45, *p* = 0.046).

**Table 8:** Logistic regression analyses with a high-quality result (final PAR score ≤5) as the dependent, binary variable; independent variables that indicated clinical relevance and/or statistical significance in univariate analyses (crosstabs) were tested as potential predictors Logistische Regressionsanalyse mit hoher Ergebnisqualität (finaler PAR-Wert ≤5) als abhängige, binäre Variable; Testung von unabhängigen Variablen als Prädiktoren, sofern nach univariater Analyse (Kreuztabellen) klinische Relevanz und/oder statistische Signifikanz anzunehmen war

		Predictive factors				
Model 1		Mean total PAR score (T0), L‑MBA, OS, RME, RA, SE, gender, age				
Model 2		Mean total PAR score (T0), RME, RA, SE				
	*N*	Predictive factors	Odds ratio	95% CI		*p*-value^a^
				Lower bound	Upper bound	
Model 2	335	Mean total PAR (T0)	0.98	0.95	1.00	0.077
		RME	0.45	0.20	0.99	**0.046**
		RA	1.92	0.89	4.14	0.098
		SE	1.27	1.11	1.46	**0.001**
		Constant	2.11	–	–	–

*CI* Confidence interval, *SE* staff experience, *RA* removable appliance, *L‑MBA* fully individualized lingual multibracket appliance, *HA* Herbst appliance, *RME* rapid maxillary expansion, *OS* orthognathic surgery, *PAR *Peer Assessment Rating Index

^a^*bold* values represent statistical significance at 5%

## Discussion

Since the aim of this study was to report about orthodontic reality in Germany, we defined very few inclusion and exclusion criteria. The age limit was chosen because we incorporated patient-reported outcomes such as oral health-related quality of life in our cohort study and therefore used questionnaires with an age recommendation. Note that these patient-reported outcomes will be part of future publications, however. Every study center was asked to consecutively screen all patients with upcoming posttreatment record takings. Yet, only an average of 57.2% of the screened patients were recruited, mostly because of a lack of patient and/or parent acceptance with regards to reading and filling-out the informed consent documents and the above-mentioned questionnaire. Although our recruitment rate and the corresponding drop-out-rate are in line with previous studies revolving around patient-reported outcomes and questionnaires [18, 34], one has to keep in mind that the present results might not fully represent orthodontic *reality* in Germany. The results should be regarded as a hint towards the *potential* quality of orthodontic care in Germany, which proved to be high within the selected study sample.

The study centers involved were chosen in order to represent orthodontic care at university hospitals as well as at orthodontic practices, yet patient and treatment characteristics along with the detected treatment outcome might not be representative for all orthodontic practitioners in Germany. Nevertheless, uniting seven national—geographically and conceptually different—study centers within this quality of orthodontic care study is unique [2, 4, 10, 21, 28, 29].

The patient characteristics within our study were similar to comparable studies: An enlarged overjet (= KIG ‘D’) was the most frequent indication to treat among the study participants. In Western Europe and especially in Germany, this occlusal feature is a frequent trait, which underlines that our study sample seems to be representative [14, 22]. More females than males made up the sample, which is characteristic for the gender distribution in orthodontics [13, 30], especially the older the patient becomes. Patient gender was not a significantly influencing factor regarding treatment effectiveness, in accordance with other studies [8, 13]. Mean age at active treatment start was 14.8 years. Quach et al. found similar gender and age distributions among their UK sample [30]. González-Gil-de-Bernabé et al. reported about older patients within their Spanish sample—17 years—[13], while Freitas et al. analyzed data from patients who were 13.5 years old on average [11]. Depending on the research question and methodology—analyses of specific treatment modalities or analyses of quality of care in general—there is no consistency in international literature about the mean age at the beginning of orthodontic treatments. With regards to patient age, we found a significantly different distribution within the PAR categories ‘greatly improved’/’improved’/’worse or different’: On the one hand, patients with great improvement were 0.6 years younger than patients who achieved mere improvement. On the other hand, these patients were older than the patients who finished with an improvement less than 30%. Thus, according to our data, there is no clinically relevant conclusion regarding the correlation between patient age at active treatment start and occlusal outcome.

Average active treatment duration within this sample was 31.3 months; active MBA treatments lasted 26.2 months on average. This is comparable to other studies that reported about treatment duration [2, 3, 10, 11, 40]. There are many factors that may potentially influence treatment duration in orthodontics, for example, the treatment modality or the need for orthognathic surgery [26]; other influencing factors are individual occlusal traits like impacted canines. Our sample comprised almost every aspect of malocclusion and treatment modality because the aim of this study was to report about orthodontic reality with its numerous facets. Although we found a significantly different distribution of treatment duration within the PAR categories of improvement—namely that the treatment duration was longer in the ‘greatly improved’ group than in the ‘improved’ group but shorter than in the ‘worse or no different’ group—no clear conclusion can be drawn about the influence of treatment duration with regards to occlusal outcome. Yet, one has to keep in mind that unwanted side effects for oral soft and hard tissues might be more probable in prolonged orthodontic treatment. Therefore, active treatment duration should be as long as necessary and as short as possible.

In general and regardless of potential confounding factors, the PAR score reduction within this German sample indicated a high standard of orthodontic care. An improvement rate higher than 70% is generally considered a good standard of orthodontic treatment [32]. In our sample, a mean PAR improvement rate of 83.54% was achieved, which seems to be rather high compared to the reported improvement in similar studies [2, 3]. Freitas et al. reported about 78.54% improvement in their Brazilian sample that underwent premolar extraction treatments [11]. Ponduri et al. investigated the PAR Index reduction of orthodontic as well as orthognathic surgery treatments and found an improvement rate of 77 and 74%, respectively [29]. An improvement rate about as high as in our sample was reported by Isherwood et al. [16] and Onyeaso et al. [28]. Moreover, the distribution of cases within our sample with regards to the PAR categories ‘greatly improved‘ (49.0%), ‘improved’ (50.1%), and ‘worse or no different’ (0.9%) represents a very high standard of treatment. Although other study groups have reported about negligible numbers of treatments that resulted in a ‘worse or no different’ outcome as well, 0.9% of ‘worse or no different’ cases, like in our sample, seems to be a very low proportion [2, 3]. On the other hand, researchers like Ponduri et al. [29] and Isherwood et al. [16] even reported about no single ‘worse or no different’ treatment within their samples. Within the present study sample, all PAR components significantly improved throughout treatment course. Yet, improvement rate of the PAR components ‘buccal segments’ and ‘centerline’ was only 44.02 and 33.23%, respectively, whereas the PAR component ‘upper anterior segments’ showed an improvement of 90.94%. These differing PAR component-specific improvement rates are in line with previous studies [2, 10, 37]. Interestingly, in the present sample the PAR component ‘buccal segments’ was reduced by 52.86% in the high SE group and only by 36.92% in the low SE group (*p* < 0.001), highlighting the potential impact of staff experience.

While many authors investigate *improvement* measures with regards to the PAR Index, few report about the final occlusal *result* as an indicator of treatment quality. Quach et al. expressed the importance of this indicator [30]. Mere improvement measures should be read with caution because the initial PAR score seems to be highly relevant and influential for the categories of PAR Index improvement (‘greatly improved’, ‘improved’, ‘worse or no different’), having in mind that a case only classifies for ‘great improvement’ when the initial PAR score counts more than 22 points. Thus, Quach et al. had a closer look at the final occlusal outcome and the percentage of treatments that finished with an almost ‘ideal occlusion’ of ≤5 PAR points; 67.9% of the 495 treated and analyzed patients from the UK had such an almost ‘ideal occlusion’ at the end of the treatment, while the improvement rate was 80.5% [30]. The 335 analyzed patients from our study were treated towards high-quality results more frequently; 81.5% fell in this outcome category, again an indicator of a very high standard of orthodontic care in this German sample. Remarkably, even if a final PAR score of 5 points or less stands for an almost ‘ideal occlusion’ and a high-quality treatment result, this low score might as well comprise a bilateral single tooth crossbite, for example.

In specific, several treatment modalities were significantly more often associated with ‘great improvement’ and high-quality treatment outcome than others. Yet, based on the aims and design of the study, no scientific explanation for the difference in appliance performance can be given. Treatments with HA resulted in high-quality treatment outcome, as it has been proven before [4]. Furthermore, treatments with L‑MBA were associated with high effectiveness. Note that most of the HA treatments and all L‑MBA cases of this sample were treated in specialized practices/university hospitals, which might be a biasing factor regarding the quality of treatment outcome. A recent systematic review on lingual orthodontics came to the conclusion that especially individualized treatment goals seemed to be achievable by fully customized L‑MBA such as those used in the present sample [27]. In addition, the PAR component ‘buccal segments’ was significantly more improved in the present L‑MBA group compared to all other treatments, possibly due to their biomechanical properties.

While patients who were treated with RME appliances had only half the chance of achieving an almost ‘ideal occlusion’ compared to the rest (OR 0.441), this negative correlation was not the case with regards to achieving the PAR category ‘greatly improved’. Significantly more patients with RME classified for ‘great improvement’ in comparison to the rest (78.6 vs. 44.9%), while significantly less patients who underwent RME treatment achieved an almost ‘ideal occlusion’ in comparison to the rest (66.7 vs. 83.9%). This apparent contradiction reflects the above-mentioned difference of the two outcome measures ‘high-quality treatment result’ and ‘great improvement’. A case can be regarded as ‘greatly improved’ because of a reduction of the mean PAR score of >22 points, but it does not necessarily finish with an almost ‘ideal occlusion’ with ≤5 PAR points at the end of treatment. RME is used when treating crossbites and a compromised transverse occlusion. In general, especially the PAR component ‘buccal segments’ proved to finish with rather high PAR points. Therefore, it seems to be clinically comprehensible that RME treatments do not finish with a low total PAR score, but can be regarded as ‘greatly improved’ nevertheless. Our result that RME treatments significantly reduce the chance to achieve high-quality treatment results should not lead to direct clinical implications or restrictions, particularly because of the above-mentioned thoughts.

Similar results were found when analyzing the effectiveness of OS treatments compared to the rest. Significantly more patients who underwent orthognathic surgery classified for ‘great improvement’ in comparison to the rest (80.0 vs. 47.1%), while significantly lower percentage of patients who were treated with surgery achieved an almost ‘ideal occlusion’ in comparison to the rest (65.0 vs. 82.8%). These findings could be explained by the previously mentioned difference between both outcome measures, but should be regarded with caution because the subgroup of OS compromised only 20 patients, while the rest made up 314 patients within the mentioned analyses.

Based on our results, a key predictive factor for finishing with an almost ‘ideal occlusion’ was a high staff experience in years. This result is not surprising and supported by other studies [10, 11, 30]. High staff experience and an increased skill level is likely to come along with increased treatment effectiveness, especially with regards to achieving one of the most important and at the same time most challenging orthodontic goal—to correct buccal occlusion. However, this result should encourage university hospitals with a high number of postgraduates and rather low staff experience, to take care of sufficient supervision by highly experienced staff members, so that the difference in clinical experience and skills does not necessarily have an effect on treatment quality.

Furthermore, the results of this study imply a trend, indicating that a combination of removable—mostly functional—and fixed appliances might result in high-quality treatment outcomes. Whenever treatments were carried out with a RA/functional phase, the chance to finish with a PAR score ≤5 points was almost twice as high (OR 1.92). Yet, this result should only be interpreted as a trend because of missing statistical significance. Quach et al. found a similar correlation between the combination of functional plus fixed appliances and high effectiveness [30]. However, treating patients with removable plus fixed appliances prolongs the total treatment duration. As orthodontists we try to treat our patients as quickly and efficiently as possible, but should also take the above-mentioned findings into account during treatment planning.

There are several limitations of the present study with some of them already mentioned. Although recruitment procedures were discussed with professionals from the Clinical Trials Center of the University Hospital of Cologne, a positive selection of potential study participants and with it, a selection bias, cannot be completely ruled out. However, a willful (positive) selection of patients was defined as unacceptable and existing good clinical practice guidelines as well as the individual commitment of research partners should not be doubted in general. Study centers with a rather long recruitment duration did not prove to provide study participants whose treatments were more efficient than the rest; in fact, the duration of recruitment was rather short at study centers that contributed a large amount of high-quality treatments. Yet, we cannot be certain about the generalizability of data. Especially with regards to specific treatment modalities such as L‑MBA or HA it is important to keep in mind that highly specialized centers were part of this study. In addition, the study sample comprised a large number of patients, which was not necessarily the case for the analyzed subgroups. Thus, results revolving around treatment modalities with only a small number of patients should be read with a degree of caution. In addition, not every potential aspect of the variety of orthodontic treatment modalities was analyzed. We chose the treatment modalities carefully with regards to frequently applied procedures, yet some aspects of orthodontic reality, like aligner treatments, might be missing. Another characteristic of our methodology was the use of the PAR Index for measuring treatment effectiveness. Although this index can be regarded as the gold standard for measuring treatment effects, there are some PAR-specific aspects to keep in mind. One of them is that the PAR Index is preferred in permanent dentition cases and often scores higher in these cases than in mixed-dentition cases. Another crucial aspect is the above-mentioned fact that a final PAR score of 5 points might be far from representing a truly *ideal* occlusion. Using the PAR Index, very good insight into the quality of orthodontic care within a sample is obtained, but it does not represent the absolute occlusal truth—as no occlusal index does. Finally, when looking at our results in detail, one has to keep in mind that T1 was not directly after active orthodontic treatment, but rather after a retention phase at the time of final record taking. Furthermore, the time interval between the end of active treatment and record taking varied considerably (6.3–26.9 months) between the study centers. Potential relapse or further improvement due to specific retention protocols were not accounted for.

In addition to the above-mentioned and -discussed findings, patient-reported outcomes were measured and analyzed in the course of this multicenter cohort study. This specific aspect of quality of orthodontic care will be part of future publications.

## Conclusion

The improvement rate among this selected German cohort indicates an overall very good standard of orthodontic care. Staff experience proved to be a predictive factor for high-quality treatment.
